# Supplementary material for: The causal relationship between the human gut microbiota and pyogenic arthritis: a Mendelian randomization study
Source: Front Cell Infect Microbiol. 2024 Nov 26;14:1452480. doi: 10.3389/fcimb.2024.1452480 (PMC11629706; doi:10.3389/fcimb.2024.1452480)
Supplement: Supplementary file 1 [file DataSheet1.zip › mendelian test/ebi-a-GCST90027608.csv_leave_one_out.pdf]

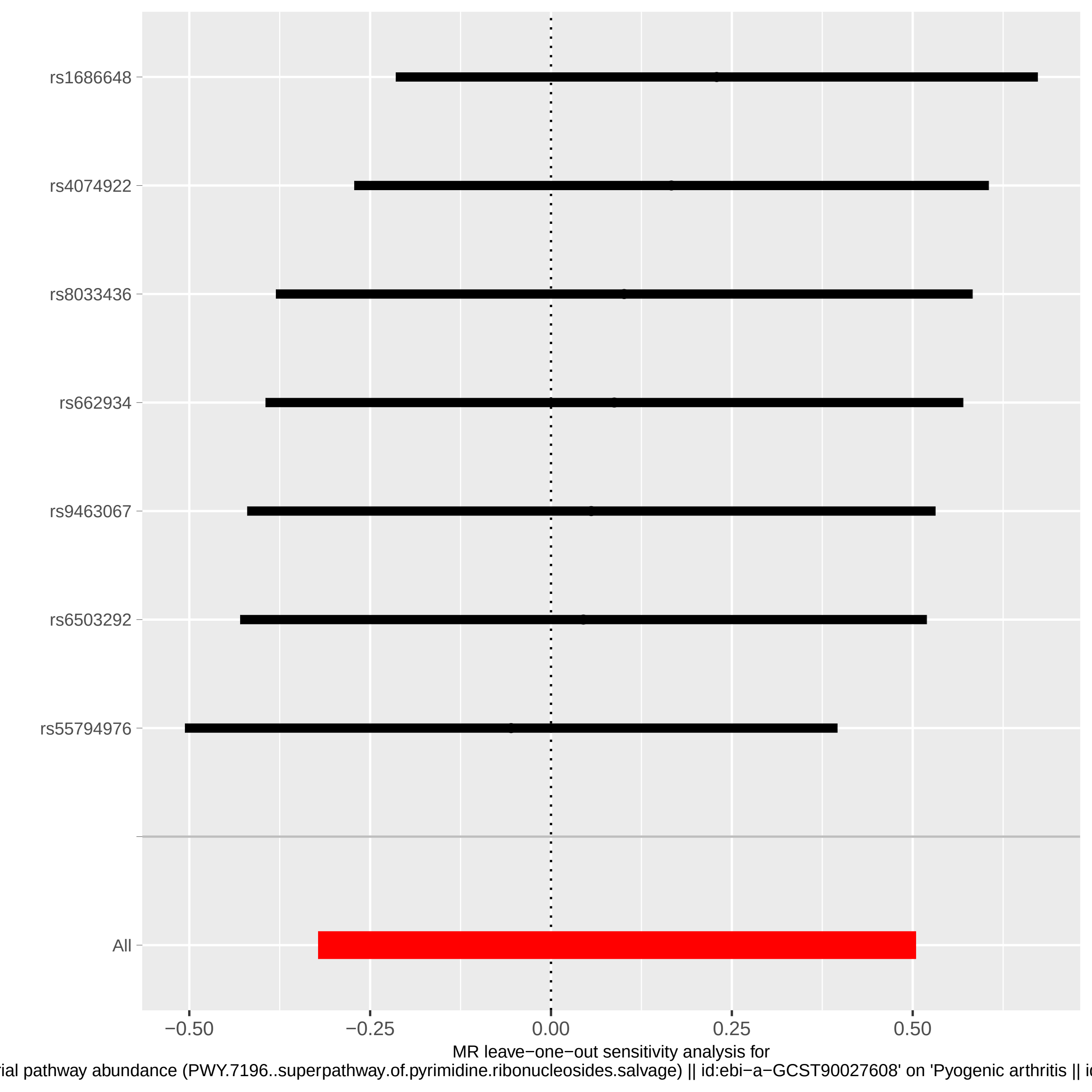

rs1686648
rs4074922
rs8033436
rs662934
rs9463067
rs6503292
rs55794976
All
−0.50
−0.25
0.00
0.25
0.50
MR leave−one−out sensitivity analysis for
rial pathway abundance (PWY.7196..superpathway.of.pyrimidine.ribonucleosides.salvage) || id:ebi−a−GCST90027608' on 'Pyogenic arthritis || i
